# Cox Models for Ecologic Time-Series Data?

**DOI:** 10.1289/ehp.114-1764157

**Published:** 2006-12

**Authors:** Thomas Lumley, Holly Janes, Lianne Sheppard

**Affiliations:** Department of Biostatistics, University of Washington, Seattle, Washington; Department of Biostatistics, Johns Hopkins Bloomberg School of Public Health, Baltimore, Maryland, E-mail: hjanes@jhsph.edu; Department of Occupational and Environmental Health Sciences, University of Washington, Seattle, Washington

In a recent article, Lepeule et al. (2006) proposed using Cox regression with time-dependent covariates to estimate the acute health effects of air pollution. Their results were similar to those they obtained in a previous case-crossover analysis (Filleul et al. 2004), and they claimed that the Cox model approach is more precise. Understanding their results and why the claim is misleading requires considering how case-crossover and Cox model analyses work.

The case-crossover design (Maclure 1991) requires a choice of referent strategy or a method for choosing control time periods (referent windows). With a valid referent strategy—a localizable design (Janes et al. 2005a; Janes et al. 2005b)—a conditional likelihood is constructed by conditioning on the number of events experienced by each person over the study period. Conveniently, there is no information on the exposure effect from people who do not have an event, so no information is lost by dropping them from the analysis. The information comes from variations in exposure within person and within referent window. We must assume that all variables that confound the variation in risk within an individual across a referent window have been measured. The estimated β is the value that equates the exposure on the index day to its expected value over the referent window, averaged over all subjects.

The Cox model (Cox 1972) uses the same principle of equating the observed and expected exposure, but across people rather than within a person. Time points with no events do not contribute information for estimating the exposure effect and may be discarded. The information comes from comparisons between people at the same point in time. We must assume that all variables that confound variation in risk between individuals at the same point in time have been measured. The estimated β is the value for which the exposure for the person with the event equals its expected value over the at-risk cohort, averaged over all time points.

If the same time scale is used for the case-crossover and Cox analyses, the two sets of information do not overlap: the case-crossover analysis is purely within person; the Cox model analysis is purely between persons. When exposure measurements vary both over time and by individual, the two analyses provide independent estimates of risk. In a data set that includes only chronic exposure measurements, there is no temporal exposure variation so the Cox model captures all the information. Conversely, in an ecologic time-series data set, there is no variation in exposure between people at a given time; therefore, the case-crossover analysis uses all of the information.

In order to estimate acute effects with ecologic exposure measurements using a Cox model, Lepeule et al. (2006) used age as the time scale. That is, they chose β, so that the exposure for an individual who died at a given age is equal to the average exposure for at-risk individuals at exactly that age. Because all individuals have the same exposure measurement on any given day, this is equivalent to comparing exposure on the day of death with exposure on a selected set of other days determined by the dates other members of the cohort reach that age. That is, it is a case-crossover design, albeit one with an unusual choice of referent strategy. Note also that the Cox regression estimating equations are exactly the same as those used in conditional logistic regression, making the case-crossover and Cox regression estimates identical.

This Cox model approach is a case-crossover design. Theoretical development is needed to determine whether it is a localizable design. It is more effcient than a semisymmetric bidirectional case-crossover design only because more referent time points are used.

We see at least two potential biases associated with this design. First, it is not clear that the strong seasonality and time trends in air pollution and mortality data are controlled with this referent strategy; typically, referent windows are designed to be small to control for time-dependent confounders by design. This referent strategy necessitates controlling such factors by modeling, as these authors have done. Second, there may be minor bias due to subjects who die very young or very old being dropped from the analysis because they have no referents (no one else is at risk at that age).
